# Opto-thermally excited multimode parametric resonance in graphene membranes

**DOI:** 10.1038/s41598-018-27561-4

**Published:** 2018-06-19

**Authors:** Robin J. Dolleman, Samer Houri, Abhilash Chandrashekar, Farbod Alijani, Herre S. J. van der Zant, Peter G. Steeneken

**Affiliations:** 1 Kavli Institute of Nanoscience, Delft University of Technology, Lorentzweg 1, 2628 CJ Delft, The Netherlands; 2 Department of Precision and Microsystems Engineering, Delft University of Technology, Mekelweg 2, 2628 CD Delft, The Netherlands; 3 Present Address: NTT Basic Research Laboratories, NTT Corporation, 3-1, Morinosato Wakamiya, Atsugi, Kanagawa 243-0198 Japan

## Abstract

In the field of nanomechanics, parametric excitations are of interest since they can greatly enhance sensing capabilities and eliminate cross-talk. Above a certain threshold of the parametric pump, the mechanical resonator can be brought into parametric resonance. Here we demonstrate parametric resonance of suspended single-layer graphene membranes by an efficient opto-thermal drive that modulates the intrinsic spring constant. With a large amplitude of the optical drive, a record number of 14 mechanical modes can be brought into parametric resonance by modulating a single parameter: the pre-tension. A detailed analysis of the parametric resonance allows us to study nonlinear dynamics and the loss tangent of graphene resonators. It is found that nonlinear damping, of the van der Pol type, is essential to describe the high amplitude parametric resonance response in atomically thin membranes.

## Introduction

The history of parametric oscillations dates back to the 19th century and the observation of surface waves in the famous singing wineglass experiment of Michael Faraday^1^. A mechanical system can be parametrically excited when its stiffness is modulated at a frequency of 2*ω*_0_/*n*, where *ω*_0_ is the system’s resonance frequency and *n* an integer^2^. Above a certain modulation amplitude, the system becomes unstable and exhibits parametric resonance. The advent of micro and nano engineering brought to life new ideas for exploiting parametric excitation for enhancing force and mass sensitivity^2–8^, effective quality factor^9^, and signal to noise ratio^3^ of tiny resonators. To date, many sensors, including gyroscopes^10–12^, mass sensors^6–8^ and even mechanical memories^13–16^ employ parametric excitation for improved performance.

Resonators employing two-dimensional materials such as graphene or molybdenum disulfide have attracted considerable interest in the scientific community^17–20^. They are promising candidates for various sensing applications^21–26^ due to their ultra high surface to mass ratio, combined with their high strength^27^. However, the quality factor of resonance is relatively low compared to other nano-electromechanical systems^18,20,28^, limiting their accuracy as a resonant sensing element. It is thus of interest to apply parametric amplification schemes to raise their effective quality factor and improve their performance. Several works have successfully demonstrated such an amplification scheme by applying an electrostatic spring force to the membrane and modulating its strength^29,30^. It is well known that above a certain critical force of this parametric pump, the device will become unstable and exhibit parametric resonance^2,31,32^. Although such behavior has been previously observed, the nonlinear dynamics involved in parametric resonance has received less attention. We demonstrate that parametric resonance holds important information about the nonlinear damping of graphene that has been a subject of strong debate in the community^28,33–36^.

In this work it is demonstrated that opto-thermal tension modulated single layer graphene is an ideal system to study parametric resonance. Despite the relatively low Q-factor of the graphene resonances (<1000), it is shown that a record number of 14 modes can be brought into parametric resonance^2,14^. The origin of the effectivity of graphene for parametric resonance is the large tension modulation that can be achieved by opto-thermal means^20,22^, which is related to the large Young’s modulus of graphene. Understanding parametric resonance is of fundamental interest, but also provides an interesting alternative to direct excitation in future applications, that could reduce noise and facilitate large amplitude driving in resonators and oscillators. The parametrically excited nonlinear mechanical response is analyzed and a model is proposed that can simulate both parametric and directly driven responses. This nonlinear Duffing response, caused by the direct drive, has previously been studied to obtain the stiffness properties of graphene devices^37,38^. In this work, we focus the analysis on dissipation mechanisms in graphene. Period doubling bifurcations are almost fully governed by the linear dissipation terms, while the saddle node bifurcation of the parametric resonance is fully governed by nonlinear dissipation terms^32^. From this analysis, we can conclude that nonlinear damping in graphene can be accurately described by a dissipation term of the van der Pol type. Comparing this to the cubic stiffness term allows us to extract the mechanical loss tangent of graphene which is orders of magnitude larger than expected. Tracking the period doubling bifurcations shows that the region of instability is asymmetric. This unexpected deviation from the theoretical response suggests that unconventional dynamic phenomena are governing the linewidth of graphene resonators.

## Experimental Setup

**Figure 1:**
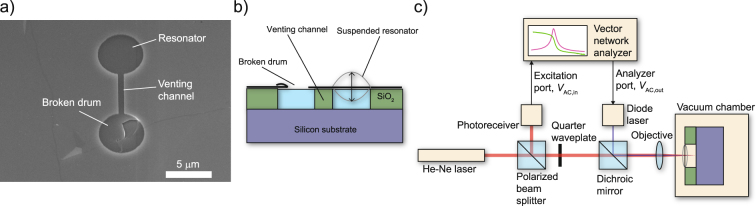
Single layer graphene resonators and the experimental setup. (**a**) Single layer graphene resonator under a scanning electron microscope (SEM). (**b**) Cross section of the device (not to scale). (**c**) Schematic of the measurement setup to actuate the membrane thermally and detect its motion by interferometry.

Experiments are performed on single-layer chemical vapour depositied (CVD) graphene drum resonators with a diameter of 5 *μ*m and a cavity depth of 300 nm. The drums have venting channels to the environment to prevent the trapping of gas in the cavity (Fig. 1(a,b)), see Methods section for details on the fabrication). To achieve parametric drive, we use the experimental setup shown in Fig. 1(c). The light from a blue diode laser is focused on the membrane and its intensity is modulated by an input voltage *V*_ac,in_. This periodically heats up the membrane and creates a parametric drive due to the thermal strain. Parametric resonance occurs if the parametric driving term *δ* exceeds a threshold *δ*_*t*_, determined by the resonance frequency *ω*_0_ and quality factor *Q* of resonance^3^. Below the threshold, the parametric drive can be used for amplification, experiments on this are shown in Supplementary Information S3. Imperfections such as initial out-of-plane deformations, wrinkles and ripples in the membrane geometry enable the blue laser to directly drive the resonator by thermal expansion force, because thermal expansion will enhance these deformations and thus actuate the membrane. A more detailed discussion on this mechanism can be found in the Supplementary Information S4.

A red helium-neon laser is used to read out the motion by the optical interference between the graphene membrane and the fixed substrate^17,20,39^. The ratio of the AC voltage amplitude generated by the photodetector and the AC driving voltage of the blue laser *V*_ac,out_/*V*_ac,in_ is determined by a vector network analyzer (VNA). Homodyne and heterodyne detection can be performed in this setup, such that both direct and parametric resonances can be analyzed.

## Multi-Mode Nonlinear Resonance

**Figure 2:**
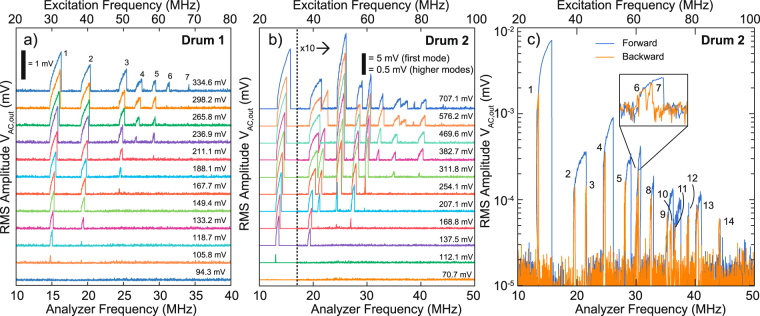
Multi-mode response of a parametrically driven graphene resonators. (**a**) Waterfall plot of the multimode response at different driving amplitudes. Each mode appears at different driving levels due to variations in quality factor and effective driving force between them. The scale bar indicates the root mean square value (RMS) of *V*_ac,out_ and the labels on the right indicate the RMS driving amplitude *V*_ac,in_. (**b**) Waterfall plot for a different drum, showing more mechanical modes and modal interactions. (**c**) Forward and backward frequency sweep at the highest parametric driving amplitude for the drum in (**b**) revealing 14 distinct mechanical modes in parametric resonance.

In Fig. 2(a), the blue laser is driven at 2 *f*, while detecting the photodiode signal at *f*. When increasing the blue laser driving voltage *V*_ac,in_ a remarkable effect is observed. One-by-one, the parametric resonances of graphene appear, up to 7 different modes. Each mode reaches resonance at a different threshold driving amplitude *V*_ac,in_, due to differences in quality factor and the frequency dependence of the parametric driving parameter *δ*^40^. The experiment is repeated on a different drum in Fig. 2(b). Interestingly, in this case overlap between parametric resonances is observed at high driving levels. When overlap occurs, a direct transition between the high-amplitude solution of two adjacent parametric resonances is observed, e.g. at *V*_ac,in_ = 382.7 mV (RMS) between the second and third resonance. Moreover, in some cases transitions between the high-amplitude and low-amplitude solutions are observed, e.g. at *V*_ac,in_ = 489.6 mV (RMS) between the same 2 modes. This random process is attributed to a strong dependence of the basin of attractions of the parametric high-amplitude and low-amplitude solutions on the initial conditions^41^. Hence, the amplitude can fall into two stable solutions: either the high amplitude solution of the third mode or the zero amplitude solution of the third mode which is also observed at higher driving amplitudes (*V*_ac,in_ = 576.2 and 707.1 mV (RMS)).

Due to the overlap of parametric resonances in this drum, some resonances are skipped and not all resonances are found by sweeping from low to high frequency. Instead, when sweeping the frequency backward as shown in Fig. 2(c), one can observe the period doubling bifurcations of the resonator. As many as 14 parametric resonances are observed in this system, whereas previously only 7 modes could be excited in cryogenic environments^14^.

**Figure 3:**
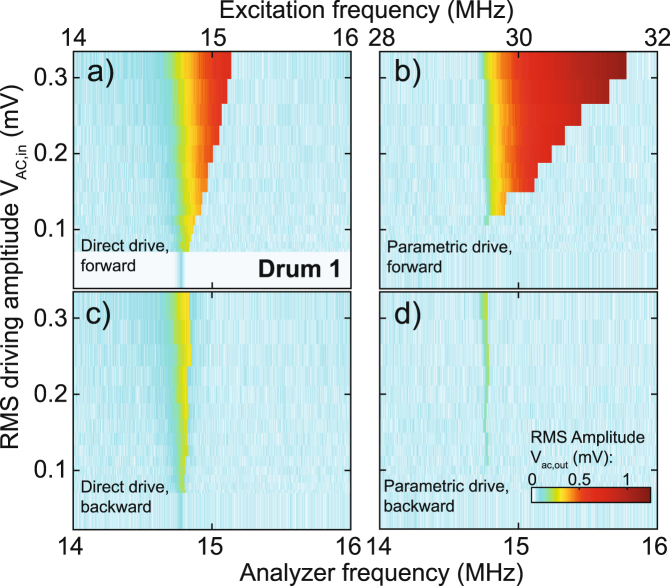
Frequency response of the fundamental mode to direct and parametric drive, for forward and backward frequency sweeps. (**a**) Direct drive with the frequency swept forwards. (**b**) Parametric drive with the frequency swept forwards. Below a driving threshold near *V*_ac,in_ ≈ 0.11 mV (RMS) no mechanical response is observed. (**c**) Direct drive with the frequency swept backwards. (**d**) Parametric drive with the frequency swept backwards.

For a more detailed analysis of the physics, we focus on the frequency response of the fundamental mode to both direct and parametric drives. Figure 3 shows direct and parametric resonance of the fundamental mode as function of driving level, on a different drum than Fig. 2. The VNA is configured to detect the directly driven frequency response (Fig. 3(a,c)). Sweeping the frequency forward (Fig. 3(a)) and backward (Fig. 3(b)) results in a hysteresis, that grows as the driving level is increased. This is typical for the geometric nonlinearity of the Duffing-type, where the stiffness becomes larger at high amplitudes. In order to detect the parametric resonance, the VNA was configured in a heterodyne scheme at which *V*_ac,out_ is detected at half of the driving frequency *V*_ac,in_. Similar to the directly driven case, a hysteresis occurs between the forward (Fig. 3(b)) and backward (Fig. 3(d)) sweeps in frequency. Below an RMS drive amplitude of 0.11 mV, *δ* < *δ*_*t*_ and no response is observed. For *δ* > *δ*_*i*_ the parametric resonance obtains two stable phases of resonance separated by 180 degrees^13^, an additional measurement that measures this behavior is shown in the Supplementary Information S2.

**Figure 4:**
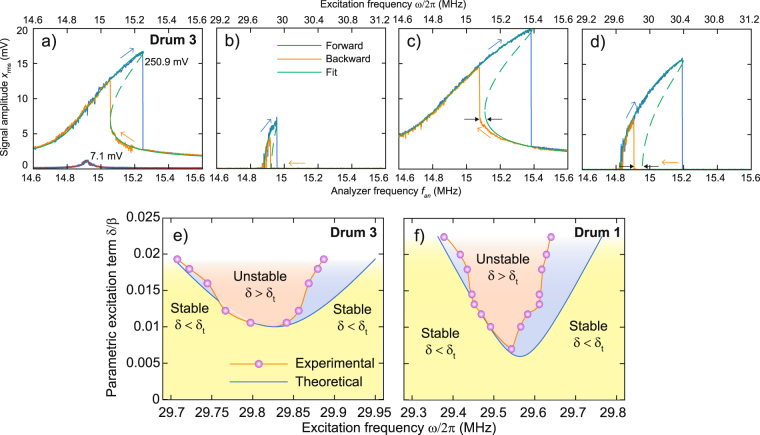
Comparison of experimental mechanical responses to theory. (**a**) Directly driven response at 7.1 and 250.9 mV RMS driving voltage and the fit obtained from Eq. 1. (**b**) Parametric response and fit at 250.9 mV RMS driving voltage and the fit from Eq. 1. (**c**) Directly driven response at 446.2 mV RMS driving level, the fit from Eq. 1 shows a disagreement with the backward sweep, highlighted by black arrows. (**d**) Parametric response at 446.2 mV (RMS). Black arrows highlight the disagreement between Eq. 1 and experiment. (**e**) Parametric resonance instability map for the fundamental mode of drum 2, compared to the prediction from Eq. 1. (**f**) Parametric resonance instability map for the fundamental mode of drum 1 (Fig. 2).

Figure 4(a–d) shows both directly and parametrically driven responses at different driving levels. In order to eludicate the effect of nonlinearities on the observed mechanical responses, a single degree-of-freedom model is derived that describes the motion of the resonator (see Supplementary Information S5) and this is fitted to the response curves in Fig. 4(a–d) (see Supplementary Information S6). The model is a combination of the Duffing, van der Pol and Matthieu-Hill equation also used in other works^32,34,42,43^:

1 $$\ddot{x}+\mu \dot{x}+\nu {x}^{2}\dot{x}+(\beta +\delta \,\cos \,\omega t)x+\gamma {x}^{3}=F\,\cos \,\omega t,$$

where *x* is the displacement (which is approximately proportional to *V*_ac,out_), *μ* is the damping coefficient, *ν* the nonlinear damping coefficient, *β* the linear stiffness coefficient, *γ* the nonlinear stiffness coefficient, *δ* cos *ωt* the parametric driving and *F* cos *ωt* the direct driving term. By setting *γ* = 0 and *ν* = 0 one can fit the direct response at low drive level (Fig. 4(a)) which is used to obtain values for *μ*, *β* and *F*. Then, *γ* is used to fit the large amplitude direct response in Fig. 4(a,c). Finally it is found that the large amplitude parametric resonances in Fig. 4(b,d) can only be fitted using a non-zero value of the nonlinear damping term *ν*. Numerical values for the fit parameters are provided in the Supplementary Information S1.

Figure 4 compares the fitted model and the experimental data for the directly- and parametrically driven fundamental resonance. This shows excellent agreement at lower driving levels (Fig. 4(a)). We note that the fitting parameters *μ*, *ν*, *β* and *γ* are the same in the direct and parametric response within the error of the fitting procedure (see Supplementary Information S1) and that both *δ* and *F* are nearly proportional to driving voltage *V*_AC,in_. The region of instability (Fig. 4(e,f)) is constructed by tracking the experimentally observed with the fitted values of the period doubling bifurcations as function of the driving parameter *δ*/*β*, which was extracted from the fit. It is observed that the region of instability is narrower and assymetric in our experiments than what is expected from Eq. 1.

## Region of Instability

The asymmetry around *ω*_0_ observed in the region of instability (Fig. 4) is a surprising result: such an asymmetry should not arise for the equation of motion (Eq. 1) used in the analysis. Something similar is observed in the directly driven response, where the lower saddle node bifurcation in the downward frequency sweep is always found at a lower frequency than simulated (Fig. 4(c)) at high driving levels. Possibly, this indicates that the excitation terms are nonlinear^44^. However, we find that both forcing terms *δ* and *F* extracted from the fits are linear with the applied modulation amplitude and the forward frequency sweeps are well-described by this model (see Supplementary Information S1). Also taking into consideration that the membrane reaches instability from a flat configuration (thus at low amplitudes), the observed deviations (e.g. in Fig. 4) can therefore not be explained by nonlinearities in the excitation.

The asymmetry and apparent decrease in resonance linewidth (Fig. 4) thus suggest that a more sophisticated model than Eq. 1 should be considered to describe the observed effect. For instance, deviations from conventional dissipation models have been previously found in multi-layered graphene resonators^36^, where it was concluded that the van-der-Pol term $\nu {x}^{2}\dot{x}$ does not describe the nonlinear damping. Here we conclude that the van-der-Pol term is in agreement with the experiment, since it describes the saddle node bifurcation of the parametric resonances well. However, additional dissipation or stiffness terms might be needed to account for the asymmetry and narrowing of the parametric stability region (Fig. 4(e,f)).

## Mechanical Loss Tangent

The fit to the nonlinear response of the membrane allows us to extract a number for the Duffing (*γ*) and van-der-Pol terms (*ν*) in our resonators. As shown in the Supplementary Information S7, the mechanical loss tangent of graphene tan *δ*_*l*_ at the resonance frequency can be determined from the ratio of these terms, tan *δ*_*l*_ = *ν*/*γ*. From the values of the fits we obtain tan *δ*_*l*_ = 0.34 for drum 2 and tan *δ*_*l*_ = 0.15 for drum 3. The values of these loss tangents are in the same range as found by Jinkins *et al*.^45^. The obtained values for the loss tangent are relatively high for a bulk crystalline material, therefore the observed loss tangent is likely dominated by effects related to the atomic thickness of graphene, such as thermodynamic fluctuations^46^, sidewall adhesion^47^ or unzipping of wrinkles^48^.

## Discussion

Here we discuss the efficiency of the tension modulation for parametric excitation of the graphene membrane. The tension modulation Δ*n*_0_(*t*) is given by Δ*n*_0_ = *αE*_2D_Δ*T*/(1 − *ν*), where *α* is the thermal expansion coefficient, *E*_2D_ the 2D Young’s modulus, *ν* the Poisson ratio and Δ*T* the temperature modulation. Using approximate values from literature^27,49^, one finds that Δ*n*_0_(*t*) ≈ 0.003Δ*T* Nm^−1^ K^−1^, which means that a temperature modulation of 1 K already results in a tension modulation of the order of the intrinsic pre-tension *n*_0_ (estimated to be between 0.003 N/m and 0.03 N/m^40^) of the graphene membranes studied here. One can define the relative shift of the resonance frequency per unit of temperature as a figure of merit for the efficiency of the opto-thermal parametric drive: $\frac{1}{{f}_{{\rm{res}}}}\frac{{\rm{\Delta }}{f}_{{\rm{res}}}}{{\rm{\Delta }}T}=0.1$ to 1 K^−1^. This estimated value for graphene is 500–5000 times larger than in other optically excited oscillators^50^. From the fits in Fig. 4 we obtain an value of $\frac{\delta }{\beta }=\frac{{\rm{\Delta }}{f}_{{\rm{res}}}^{2}}{{f}_{{\rm{res}}}^{2}}=0.0225$ in drum 1 and $\frac{\delta }{\beta }=0.0193$. Using the approximative values above, we estimate the temperature modulation Δ*T* lies between 0.13 K and 1.5 K. These moderate temperature modulations illustrate that the parametric driving scheme for graphene membranes is a very efficient method for reaching parametric resonance.

Multi-mode parametric oscillators are interesting for applications where accurate frequency tracking of multiple modes is necessary. We list here three potential applications. (1) Radio receivers in the MHz range where multiple radio channels need to be monitored and received simultaneously to maximize data rates, or to allow seamless switching between channels without having to tune the channel^51^. (2) Inertial imaging^52^, where accurate tracking of multiple resonances allows one to determine the mass, location and shape of a particle on top of a resonator, which has applications in biotechnology^53^. (3) Parametric oscillators can also be used to build a binary information and computation system^14^, where information is stored in the phase of the resonator. Multi-mode resonators have the potential of enabling parallel processing and data storage. The high resonance frequencies and relatively low Q of the graphene membranes can increase computation speed.

A unique feature of the demonstrated graphene system is that all of these modes can be simultaneously parametrically amplified via tension modulation. The use of parametric amplification therefore has the advantage that no feedback loops or special filters or actuation schemes are needed to select the desired resonance mode. Moreover, parametric amplification effectively results in an amplitude dependent gain, which can be used to generate higher output signals than with a constant gain. Moreover, since the driving frequency is double the readout frequency, parametric driving is less sensitive to cross-talk that is often hampering resolving signal detection in directly driven resonators^2^.

## Conclusions

In conclusion, we report on multi-mode parametric resonance and amplification in single layer graphene resonators by an opto-thermal tension modulation technique. It is demonstrated that the tension-dominated restoring force results in parametric excitation of multiple resonance modes in the system when the system is opto-thermally driven. The parametrically and directly driven resonances are compared to a single degree-of-freedom model based on the Duffing, van der Pol and Matthieu equations, with good agreement at low driving levels. This allows simultaneous determination of nonlinear stiffness and damping coefficients and results in a high-frequency determination of graphene’s mechanical loss tangent. Graphene resonators are thus an interesting platform to study parametric excitations and their utilization for sensors with improved performance.

## Methods

Graphene resonators are fabricated by etching dumbbell-shaped cavities in a thermally grown, 285 nm SiO_2_ layer on a silicon wafer. The etching did not fully stop at the silicon layer, resulting in cavities that are 300 nm thick. Circular membranes are formed by transfer of single layer CVD graphene (Fig. 1(a)). During the transfer process one side of the dumbbell is broken while the other side remains intact, creating a circular resonator on one side with a venting channel to the environment (Fig. 1(b)). This prevents gas from being trapped in the cavity when the pressure in the surroundings changes. In the main section of this work four identical drums with a diameter of 5 micrometer are used; results obtained on drum 1 are shown in Figs 2(a), 3 and 4(f), drum 2 in Fig. 2(b,c), drum 3 in Fig. 4(a–e). Drum 4 and 5 were used in the Supplementary Information S2 and S3, respectively. More details on the fabrication and transfer process of the drum resonators can be found in ref.^40^.

All measurements are performed at room temperature in a high vacuum environment with a pressure less than 2 × 10^−5^ mbar to minimize the effects of gas damping. The blue diode laser (Thorlabs LP405-SP10) has a wavelength of 405 nm and is biased with a 32 mA current, resulting in 0.76 mW of incident power measured before the objective. The red laser illuminates the sample with 1.2 mW of incident power (measured before the objective lens). The vector network analyzer is of type Rohde & Schwarz ZNB4 with the frequency conversion option (k4) installed. The frequency conversion option of the VNA enables both homodyne and heterodyne detection, such that both direct and parametric resonances can be detected.

### Data availability

The authors declare that all the data in this manuscript are available upon request.

## Electronic supplementary material

Supplementary Information: Opto-thermally excited multimode parametric resonance in graphene membranes

## References

[1] Faraday M (1831). On a peculiar class of acoustical figures; and on certain forms assumed by a group of particles upon vibrating elastic surfaces. Philisophical Transactions Royal Soc. (London).

[2] Turner KL (1998). Five parametric resonances in a microelectromechanical system. Nat..

[3] Rugar D, Grütter P (1991). Mechanical parametric amplification and thermomechanical noise squeezing. Phys. Rev. Lett..

[4] Karabalin R, Feng X, Roukes M (2009). Parametric nanomechanical amplification at very high frequency. Nano letters.

[5] Karabalin R (2011). Signal amplification by sensitive control of bifurcation topology. Phys. Rev. Lett..

[6] Zhang W, Turner KL (2005). Application of parametric resonance amplification in a single-crystal silicon micro-oscillator based mass sensor. Sensors Actuators A: Phys..

[7] Zhang, W. & Turner, K. L. A mass sensor based on parametric resonance. In *Proceedings of the Solid State Sensor*, *Actuator and Microsystem Workshop*, *Hilton Head Island*, *SC*, 49–52 (2004).

[8] Zhang W, Baskaran R, Turner KL (2002). Effect of cubic nonlinearity on auto-parametrically amplified resonant mems mass sensor. Sensors Actuators A: Phys..

[9] Mahboob I, Yamaguchi H (2008). Piezoelectrically pumped parametric amplification and q enhancement in an electromechanical oscillator. Appl. Phys. Lett..

[10] Oropeza-Ramos, L. A. & Turner, K. L. Parametric resonance amplification in a memgyroscope. In *Sensors*, *2005 IEEE*, 4–pp (IEEE, 2005).

[11] Hu Z, Gallacher B, Burdess J, Fell C, Townsend K (2011). A parametrically amplified mems rate gyroscope. Sensors Actuators A: Phys..

[12] Harish K, Gallacher B, Burdess J, Neasham J (2008). Experimental investigation of parametric and externally forced motion in resonant mems sensors. J. Micromechanics Microengineering.

[13] Mahboob I, Yamaguchi H (2008). Bit storage and bit flip operations in an electromechanical oscillator. Nat. Nanotechnology.

[14] Mahboob, I., Mounaix, M., Nishiguchi, K., Fujiwara, A. & Yamaguchi, H. A multimode electromechanical parametric resonator array. *Sci. Reports* **4** (2014). 10.1038/srep04448 PMC3963032 24658349

[15] Roukes, M. Mechanical compution, redux? nanoelectromechanical systems. In *Electron Devices Meeting*, 2004. *IEDM Technical Digest*. *IEEE International*, 539–542 (IEEE, 2004).

[16] Freeman M, Hiebert W (2008). Nems: Taking another swing at computing. Nat. Nanotechnology.

[17] Bunch JS (2007). Electromechanical resonators from graphene sheets. Sci..

[18] Zande AMVD (2010). Large-scale arrays of single-layer graphene resonators. Nano letters.

[19] Chen C (2009). Performance of monolayer graphene nanomechanical resonators with electrical readout. Nat. Nanotechnology.

[20] Castellanos-Gomez, A. *et al*. Single-layer mos2 mechanical resonators. *Adv. Mater*. **25**, 6719–6723 (2013). 10.1002/adma.201303569 24123458

[21] Dolleman RJ, Davidovikj D, Cartamil-Bueno SJ, van der Zant HS, Steeneken PG (2015). Graphene squeeze-film pressure sensors. Nano letters.

[22] Dolleman RJ, Cartamil-Bueno SJ, van der Zant HS, Steeneken PG (2016). Graphene gas osmometers. 2D Mater..

[23] Koenig SP, Wang L, Pellegrino J, Bunch JS (2012). Selective molecular sieving through porous graphene. Nat. Nanotechnology.

[24] Smith A (2013). Electromechanical piezoresistive sensing in suspended graphene membranes. Nano letters.

[25] Bunch JS (2008). Impermeable atomic membranes from graphene sheets. Nano letters.

[26] Sakhaee-Pour A, Ahmadian M, Vafai A (2008). Applications of single-layered graphene sheets as mass sensors and atomistic dust detectors. Solid State Commun..

[27] Lee C, Wei X, Kysar JW, Hone J (2008). Measurement of the elastic properties and intrinsic strength of monolayer graphene. Science.

[28] Barton RA (2011). High, size-dependent quality factor in an array of graphene mechanical resonators. Nano letters.

[29] Prasad, P., Arora, N. & Naik, A. Parametric amplification in mos2 drum resonator. *Nanoscale* (2017). 10.1039/c7nr05721k 29143000

[30] Mathew, J. P., Patel, R. N., Borah, A., Vijay, R. & Deshmukh, M. M. Dynamical strong coupling and parametric amplification of mechanical modes of graphene drums. *Nat. Nanotechnology* (2016). 10.1038/nnano.2016.94 27294506

[31] Eichler A, Chaste J, Moser J, Bachtold A (2011). Parametric amplification and self-oscillation in a nanotube mechanical resonator. Nano letters.

[32] Lifshitz R, Cross M (2008). Nonlinear dynamics of nanomechanical and micromechanical resonators. Rev. Nonlinear Dynamics Complexity.

[33] Croy A, Midtvedt D, Isacsson A, Kinaret JM (2012). Nonlinear damping in graphene resonators. Phys. Rev. B.

[34] Eichler A (2011). Nonlinear damping in mechanical resonators made from carbon nanotubes and graphene. Nat. Nanotechnology.

[35] Güttinger J (2017). Energy-dependent path of dissipation in nanomechanical resonators. Nat. Nanotechnol..

[36] Singh V, Shevchuk O, Blanter YM, Steele GA (2016). Negative nonlinear damping of a multilayer graphene mechanical resonator. Phys. Rev. B.

[37] Davidovikj D (2017). Nonlinear dynamic characterization of two-dimensional materials. Nat. Commun..

[38] Sajadi B (2017). Experimental characterization of graphene by electrostatic resonance frequency tuning. J. Appl. Phys..

[39] Dolleman RJ, Davidovikj D, van der Zant HS, Steeneken PG (2017). Amplitude calibration of 2d mechanical resonators by nonlinear optical transduction. Appl. Phys. Lett..

[40] Dolleman, R. J. *et al*. Optomechanics for thermal characterization of suspended graphene. *Phys. Rev. B* **96**, 165421 (2017).

[41] Sanchez NE, Nayfeh AH (1990). Prediction of bifurcations in a parametrically excited duffing oscillator. Int. J. Non-Linear Mech..

[42] Houri S (2017). Direct and parametric synchronization of a graphene self-oscillator. Appl. Phys. Lett..

[43] Aubin K (2004). Limit cycle oscillations in cw laser-driven nems. J. Microelectromechanical Systems.

[44] Rhoads JF (2006). Generalized parametric resonance in electrostatically actuated microelectromechanical oscillators. J. Sound Vib..

[45] Jinkins K, Camacho J, Farina L, Wu Y (2015). Examination of humidity effects on measured thickness and interfacial phenomena of exfoliated graphene on silicon dioxide via amplitude modulation atomic force microscopy. Appl. Phys. Lett..

[46] Sajadi, B. *et al*. Modal analysis for determining the size-and temperature-dependent bending rigidity of graphene. *arXiv preprint arXiv:1803*.04191 (2017).

[47] Bunch JS, Dunn ML (2012). Adhesion mechanics of graphene membranes. Solid State Commun..

[48] Ruiz-Vargas CS (2011). Softened elastic response and unzipping in chemical vapor deposition graphene membranes. Nano Lett..

[49] Yoon D, Son Y-W, Cheong H (2011). Negative thermal expansion coefficient of graphene measured by raman spectroscopy. Nano letters.

[50] Zalalutdinov M (2001). Optically pumped parametric amplification for micromechanical oscillators. Appl. Phys. Lett..

[51] Mansour RR, Huang F, Fouladi S, Yan WD, Nasr M (2014). High-q tunable filters: Challenges and potential. IEEE Microw. Mag..

[52] Hanay MS (2015). Inertial imaging with nanomechanical systems. Nat. Nanotechnology.

[53] Cermak N (2016). High-throughput measurement of single-cell growth rates using serial microfluidic mass sensor arrays. Nat. Biotechnology.

