# Supplementary material for: Opto-thermally excited multimode parametric resonance in graphene membranes
Source: Sci Rep. 2018 Jun 19;8:9366. doi: 10.1038/s41598-018-27561-4 (PMC6008417; doi:10.1038/s41598-018-27561-4)
Supplement: Supplementary file 1 — Supplementary Information: Opto-thermally excited multimode parametric resonance in graphene membranes [file 41598_2018_27561_MOESM1_ESM.pdf]

Supplementary Information: Opto-thermally excited multimode parametric resonance in graphene membranes

Robin J. Dolleman[1,*], Samer Houri[1,], Abhilash Chandrashekar[2], Farbod Alijani[2], Herre S. J. van der Zant[1], and Peter G. Steeneken[1,2,*]**

[1]Kavli Institute of Nanoscience, Delft University of Technology, Lorentzweg 1, 2628 CJ, Delft, The Netherlands
[2]Department of Precision and Microsystems Engineering, Delft University of Technology, Mekelweg 2, 2628 CD, Delft, The Netherlands
[*]R.J.Dolleman@tudelft.nl, P.G.Steeneken@tudelft.nl
[**]Current affiliation: NTT Basic Research Laboratories, NTT Corporation, 3-1, Morinosato Wakamiya, Atsugi, Kanagawa, 243-0198, Japan

ABSTRACT

In section S1, we show the complete dataset obtained while analyzing the nonlinearities of the graphene membrane. Section S2 shows an additional experiment that demonstrates the parametric oscillator has two stable phases and section S3 shows additional experiments on parametric amplification. In section S4, an additional discussion is added that proposes why both direct and parametric excitations are observed in the same setup. Section S5 derives the equations of motion that has been used to perform the fitting and section S6 describes the numerical simulations used for the fitting procedure. Finally in section S7 the expression for the mechanical loss tangent of graphene is derived.

S1: Complete datasets for the analysis of mechanical nonlinearities

Figure 4 in the main text shows the fitting of the nonlinear mechanical response of the resonator (drum 3). In this section the remainder of this analysis is presented and the complete dataset from the fundamental mode of drum 1 is shown (Figs. 2(a), 3, 4(f) in the main text).

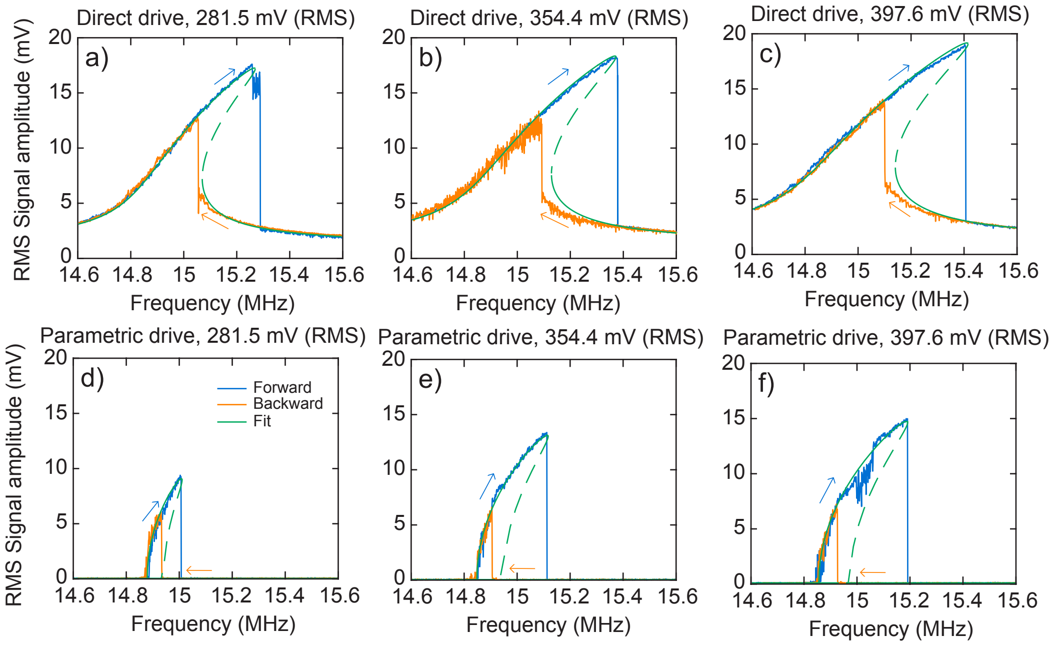

Figure S1. Remainder of the dataset presented in Figs. 4 and 5 (a-e) in the main text.

Table S1. Values obtained from the fits on the response from drum 3 in Fig. S1 and Figs. 4(a)-(e) in the main text

	Direct drive				Parametric drive			
RMS Drive (mV)	μ/β	ν/β	γ/β	$F/\beta \times 10^{-5}$	μ/β	ν/β	γ/β	$\delta/\beta \times 10^{-2}$
250.9	0.0045	70	225	8	0.0045	76	215	1.06
281.5	0.0045	72	220	9.2	0.0045	76	220	1.22
354.4	0.0045	74	230	11.7	0.0045	79	225	1.6
397.6	0.0045	76	230	14.2	0.0046	80	225	1.8
446.2	0.0045	76	230	15.5	0.0046	80	225	1.93

Dataset of drum 1

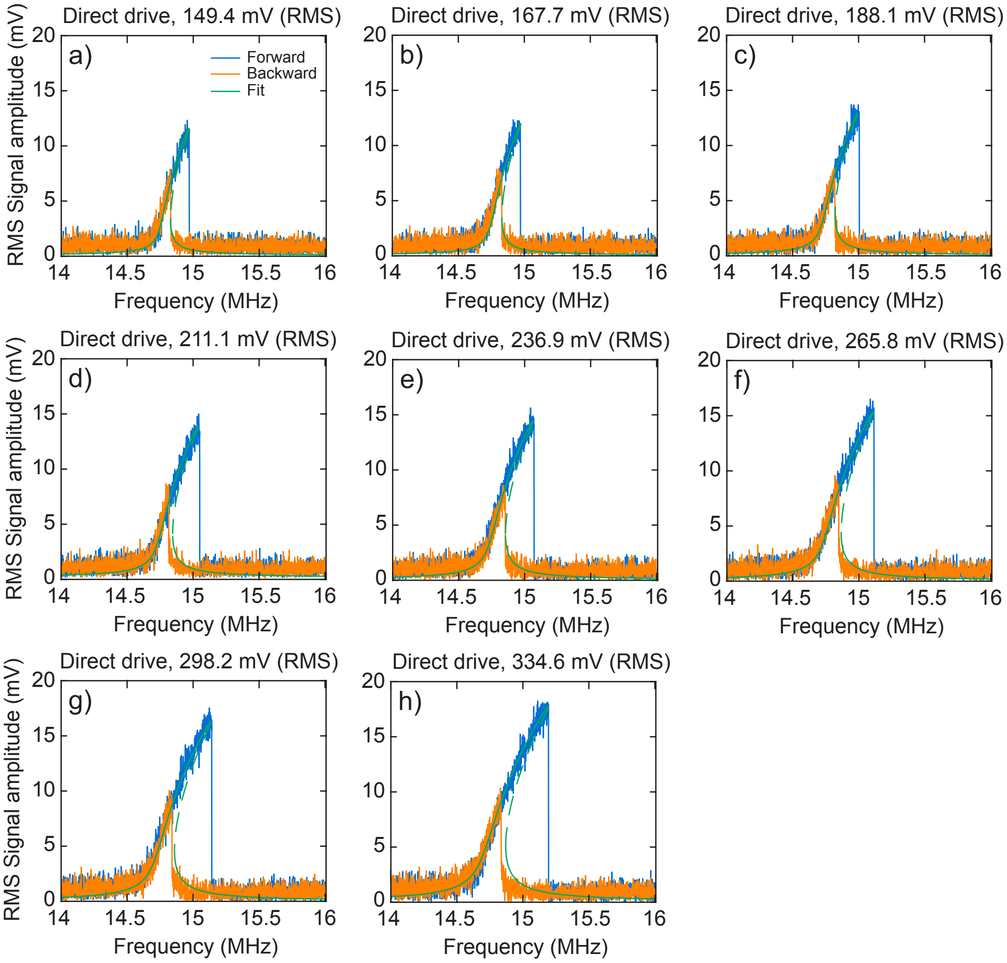

Figure S2. Analysis of directly driven response of the fundamental mode of drum 1 (Figs. 2(a), 3, 4(f) in the main text).

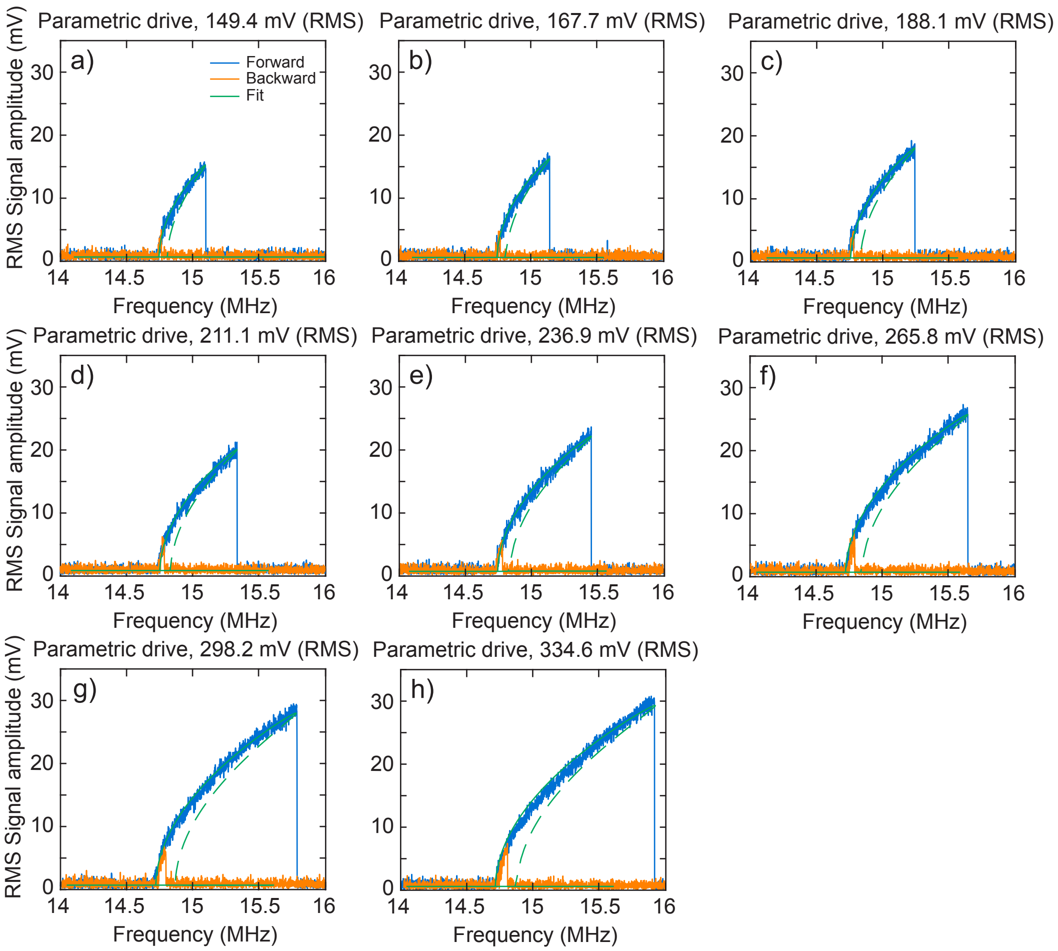

Figure S3. Analysis of parametrically driven response of the fundamental mode of drum 1 (Figs. 2(a), 3, 4(f) in the main text). The horizontal axis indicates the frequency at the analyzer port of the VNA, the frequency at the actuation port was doubled.

Table S2. Values obtained from the fits on the response from drum 1 in Figs. S2, S3 and Figs. 2(a), 3, 4(f) in the main text.

	Direct drive				Parametric drive			
RMS Drive (mV)	μ/β	ν/β	γ/β	$F/\beta\times 10^{-5}$	μ/β	ν/β	γ/β	$\delta/\beta\times 10^{-2}$
149.4	0.0030	36	250	1.42	0.0030	36	250	0.74
167.7	0.0030	37	245	1.6	0.0030	36	220	1.01
188.1	0.0030	37	245	2.0	0.0030	34	225	1.18
211.1	0.0030	37	245	2.5	0.0030	34	225	1.31
236.9	0.0030	37	250	2.8	0.0030	33	225	1.46
265.8	0.0030	36	250	3.3	0.0030	34	225	1.81
298.2	0.0030	35	250	3.9	0.0030	35	225	2.05
334.6	0.0030	35	250	4.5	0.0030	35	225	2.25

S2: Additional experiment showing two stable phases

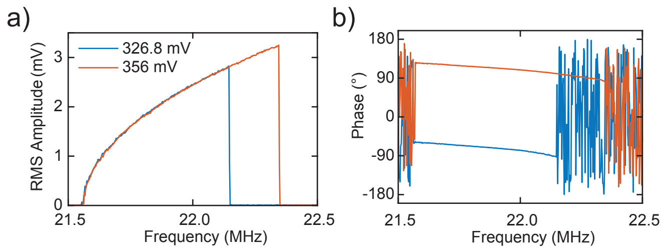

Figure S4. Additional experiment of parametric resonance. Parametric excitation was achieved using the frequency doubler (as shown in Fig. 1 in the main text) instead of the frequency conversion on the vector network analyzer. (a) Amplitude of the response at two different driving powers and (b) the phase of these repsonses. The phase shows two stable phases separated by 180 degrees as expected.

The frequency conversion option on the vector network analyzer loses information on the phase at which the resonator is oscillating. To show that the parametrically excited resonance has two stable phases separated by 180 degrees, the experiment was repeated by using the frequency doubler in the circuit used for the parametric amplification experiment (Fig. 1(c) in the main text). Using this, the VNA does not require to perform a frequency conversion and phase information is preserved. This results in the mechanical responses shown in Fig. S4.

S3: Parametric amplification

Here we investigate the effects of parametric drive at low driving levels ($\delta < \delta_t$) by examining parametric amplification of the directly driven resonance. To measure parametric amplification, it is required to simultaneously drive the system at f and $2f$ (where f is near the resonance frequency f_0). This is realized by splitting the driving circuit connected to the diode laser into two parts. One path provides a small direct drive that excites the primary resonance of the membrane in the linear regime. The second path contains a frequency doubler, amplifier and phase shifter to enable parametric driving with controllable phase and gain with respect to the direct drive. A harmonic oscillator model is fitted to the response to extract the amplitude and the effective quality factor. The relation between amplitude gain G, parametric drive amplitude δ and phase shift ϕ of the direct drive is given by[1,2]:

$$G(\delta,\phi) = \left[\frac{\cos^2\phi}{(1+\delta/\delta_t)^2} + \frac{\sin^2\phi}{(1-\delta/\delta_t)^2}\right]^{1/2}. \tag{S1}$$

First, the amplification effect as function of parametric pumping amplitude in Fig. S5(a) was examined by keeping the phase ϕ fixed at $\phi =$ -45 degrees. Increasing the amplitude of parametric drive increases the amplitude at resonance by a factor of 3-4 (Fig. S5(b)) and the effective quality factor of resonance by almost a factor of 3 (Fig. S5(c)). Figure S5(d) shows that shifting the phase of the parametric drive significantly changes the amplitude of harmonic resonance. Figure S5(e)-(f) shows that the gain G and effective Q-factor Q_{eff} depend strongly on the phase of the parametric drive with respect to the direct drive. Fits of the data in Fig. S5(b), (e) show that the drive and phase-dependence of the parametric amplification is in accordance with theory.

S4: Additional discussion: mechanism for direct and parametric driving

Opto-thermal driving leads to two mechanisms that can excite the resonance in the graphene resonators. Parametric drive (Fig. S6a) occurs due to the modulation of pretension $n_0(t)$ in the membrane via laser heating and thermal expansion, since the stiffness term for the out-of-plane deflection field w of the membrane is determined by the pre-tension. Parametric driving will only activate the parametric resonance if the modulation of the blue laser is near twice the mechanical resonance frequency.

As demonstrated in the main text (Figs. 3, 4), the experiments also show a direct driving component. This can be explained[3] by assuming a small initial membrane displacement w_0 from equilibrium (Fig. S6b). In graphene resonators rippling, wall adhesion or out-of-plane crumples could lie at the root of such an initial displacement.

In order to analyze the data, we will derive the equations of motion (Eq. S11) using a Lagrangian approach by including this initial deflection field. In this manner, the equations are reduced to a single-degree-of-freedom (s-dof) model that can be used to fit to the data, significantly simplifying the analysis. The derivation of this s-dof model is shown below in section S4.

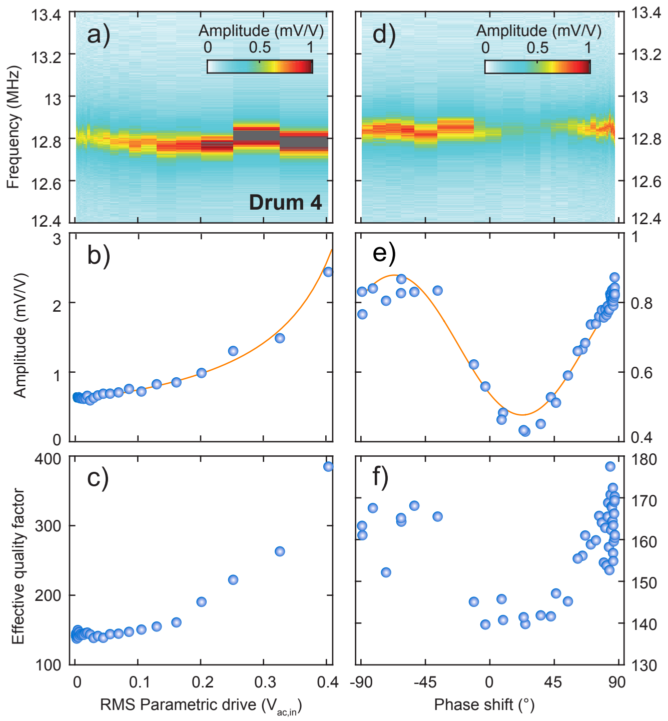

Figure S5. Parametric amplification in graphene: direct driven resonance with a sub-threshold ($\delta < \delta_t$) parametric drive. (a), Transmission function of the direct drive as function of parametric drive. (b), Amplitude of resonance obtained from a fit to a harmonic oscillator model as function of parametric drive, the red line is a fit to the theoretical behavior predicted by Eq. S1. (c), Effective quality factor, obtained from a fit to a harmonic oscillator model, as function of parametric drive. (d), Transmission funciton as function of phase shift ϕ. (e) Amplitude of resonance as function of phase ϕ, the red line is a fit using eq. S1. (f), Effective quality factor as function of phase ϕ.

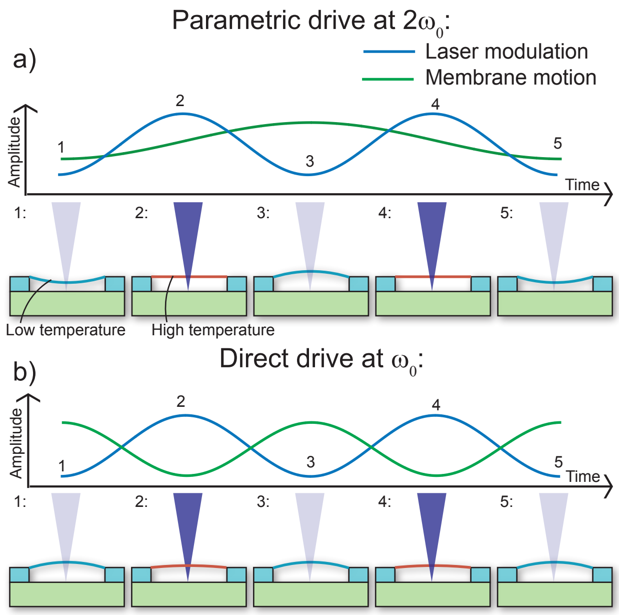

Figure S6. Explanation of the actuation mechanisms of the opto-thermal drive. For illustration of the mechanism, it is assumed the membrane motion follows the force adiabatically (phase delays are omitted). A blue membrane represents low temperature and a red membrane represents high temperature. a) Parametric excitation, this is due to the pre-tension modulation of the membrane. Each time the tension is maximum the membrane passes through its equilibrium position, leading to a period doubling. This mechanism activates the resonance if the driving frequency is twice the resonance frequency. b) Direct excitation, which exists due to a small initial deviation from equilibrium. This mechanism does not cause period doubling, but instead it activates the resonance if the driving frequency is equal to the resonance frequency.

S5: Equations of motion

A Lagrangian approach is used to obtain equations of motion of an opto-thermally excited monolayer graphene membrane. In this respect, the potential energy of the thermally actuated circular membrane is obtained as[4]:

$$U = \int_0^{2\pi}\int_0^R \frac{h}{2}\Big(\sigma_{rr}(\varepsilon_{rr}-\alpha\Delta T)+\sigma_{\theta\theta}(\varepsilon_{\theta\theta}-\alpha\Delta T)+\tau_{r\theta}\gamma_{r\theta}\Big)rdrd\theta, \tag{S2}$$

where h is the thickness, R is the radius, α is the thermal expansion coefficient, and ΔT is the temperature change in the membrane. Moreover, σ_{rr} , $\sigma_{\theta\theta}$, $\tau_{r\theta}$, are the Kirchhoff stresses that can be obtained as follows:

$$\begin{aligned}
\sigma_{rr} &= \frac{E}{1-\nu^2}(\varepsilon_{rr}+\nu\varepsilon_{\theta\theta}-\alpha(1+\nu)\Delta T),\\
\sigma_{\theta\theta} &= \frac{E}{1-\nu^2}(\varepsilon_{\theta\theta}+\nu\varepsilon_{rr}-\alpha(1+\nu)\Delta T),\\
\tau_{r\theta} &= \frac{E}{2(1+\nu)}\gamma_{r\theta},
\end{aligned} \tag{S3}$$

in which ε_{rr}, $\varepsilon_{\theta\theta}$, and $\gamma_{r\theta}$ are the Green strains and are derived as:

$$\begin{aligned}
\varepsilon_{rr} &= \frac{\partial u}{\partial r}+\frac{1}{2}\left(\frac{\partial w}{\partial r}\right)^2+\left(\frac{\partial w}{\partial r}\right)\left(\frac{\partial w_0}{\partial r}\right),\\
\varepsilon_{\theta\theta} &= \frac{\partial v}{r\partial\theta}+\frac{u}{r}+\frac{1}{2}\left(\frac{\partial w}{r\partial\theta}\right)^2+\left(\frac{\partial w}{r\partial\theta}\right)\left(\frac{\partial w_0}{r\partial\theta}\right),\\
\gamma_{r\theta} &= \frac{\partial v}{\partial r}-\frac{v}{r}+\frac{\partial u}{r\partial\theta}+\left(\frac{\partial w}{\partial r}\right)\left(\frac{\partial w}{r\partial\theta}\right)+\left(\frac{\partial w}{\partial r}\right)\left(\frac{\partial w_0}{r\partial\theta}\right)+\left(\frac{\partial w_0}{\partial r}\right)\left(\frac{\partial w}{r\partial\theta}\right),
\end{aligned} \tag{S4}$$

where u, v and w are the radial, tangential and transverse displacements, respectively. Moreover, w_0 is the deviation of the membrane from flat configuration, E is the Young's modulus and ν is the Poisson's ratio.

The temperature difference ΔT can be obtained by solving the following heat conduction equation:

$$\frac{\partial \Delta T}{\partial \tilde{t}} + \frac{\Delta T}{\tau} = \frac{P_{abs}\cos(\omega \tilde{t})}{C_t}, \tag{S5}$$

in which P_{abs} is the power absorbed by the membrane, τ is the thermal time constant[5], C_t is the thermal capacitance, and $\tilde{t}$ represents the time variable.

For a membrane with fixed edges u and w shall vanish at $r = R$. Moreover, u should be zero at $r = 0$ for continuity and symmetry. Furthermore, assuming only axisymmetric vibrations ($v = 0$ and $\partial u/\partial\theta = \partial v/\partial\theta = \partial w/\partial\theta = 0$), the solution can be approximated as[6]:

$$w = x(\tilde{t})J_0\left(\alpha_0 \frac{r}{R}\right), \tag{S6}$$

$$u = u_0 r + r(R-r)\sum_{k=1}^{\bar{N}} q_k(\tilde{t})r^{k-1}. \tag{S7}$$

Here it should be noted that for axisymmetric vibrations the shear strain $\gamma_{r\theta}$ would become zero. In equation (S6), $x(\tilde{t})$ is the generalized coordinate associated with the fundamental mode of vibration. Furthermore, in equation (S7), $q_k(\tilde{t})$'s are the generalized coordinates associated with the radial motion. Moreover, J_0 is the zeroth order Bessel function of the first kind and $\alpha_0 = 2.40483$. In addition, $\bar{N}$ is the number of necessary terms in the expansion of radial displacement and u_0 is the initial displacement due to pre-tension n_0 that is obtained from the initial stress $\sigma_0 = n_0/h$ as follows :

$$u_0 = \frac{\sigma_0(1-\nu)}{E}. \tag{S8}$$

The kinetic energy of the membrane neglecting in-plane inertia, is given by:

$$T = \frac{1}{2}\rho h \int_0^{2\pi}\int_0^R \left(\frac{\partial w}{\partial \tilde{t}}\right)^2 r dr d\theta. \tag{S9}$$

The Lagrange equations of motion are given by:

$$\frac{d}{dt}\left(\frac{\partial T}{\partial \dot{\mathbf{q}}}\right) - \frac{\partial T}{\partial \mathbf{q}} + \frac{\partial U}{\partial \mathbf{q}} = 0, \tag{S10}$$

and $\mathbf{q}$=[$x(\tilde{t})$,$q_k(\tilde{t})$], $k = 1,\ldots,\bar{N}$ is the vector containing all the generalized coordinates. Equation (S10) leads to a system of nonlinear equations comprising of a single differential equation associated with the generalized coordinate $x(\tilde{t})$ and $\bar{N}$ algebraic equations in terms of $q_k(\tilde{t})$. By solving the $\bar{N}$ algebraic equations it is possible to determine $q_k(\tilde{t})$ in terms of $x(\tilde{t})$[6]. This will reduce the $\bar{N}$+1 set of nonlinear equations to the following Duffing-Matthieu-Hill equation:

$$m\ddot{x} + c_1\dot{x} + c_2x^2\dot{x} + [k_1 + F_p\cos(\omega\tilde{t})]x + k_2x^2 + k_3x^3 = F_d\cos(\omega\tilde{t}), \tag{S11}$$

where $(\dot{\bullet})$ represents derivative with respect to time $\tilde{t}$ and m is the mass. c_1 and c_2 are the linear viscous damping coefficient and nonlinear material damping coefficient, respectively[7,8]. They are added to the equation of motion explicitly to introduce dissipation. k_1 represents the linear stiffness term dominated by the pre-tension n_0 and F_p is the amplitude of parametric drive resulting from temperature variation ΔT. Moreover, k_2 represents the quadratic nonlinear stiffness coefficient due to imperfection w_0 and k_3 denotes the cubic nonlinear stiffness coefficient arising from geometric nonlinearity. Finally, F_d is the amplitude of direct drive term due to the presence of imperfection w_0, and ω is the excitation frequency. Indeed for a flat membrane, $k_2 = F_d = 0$.

S6: Numerical simulations

In order to perform the numerical simulations, equation (S11) is normalized with respect to the mass m of the membrane and the fundamental frequency ($t = \tilde{t}\omega_0$) as follows:

$$\ddot{x} + \mu\dot{x} + \nu x^2\dot{x} + [\beta + \delta\cos(\Omega t)]x + \gamma_2x^2 + \gamma_3x^3 = F\cos(\Omega t), \tag{S12}$$

where $\beta=1$ due to the normalization. Introducing an effective stiffness nonlinearity γ, whose value is given by $\gamma=\left(\gamma_3-\frac{10\gamma_2^2}{9}\right)$[9], equation (S12) is reduced to:

$$\ddot{x}+\mu\dot{x}+\nu x^2\dot{x}+[\beta+\delta\cos(\Omega t)]x+\gamma x^3=F\cos(\Omega t), \tag{S13}$$

where the normalized coefficients are given in table S3.

Definition	Normalized parameter
$(\dot{\bullet})=\frac{d(\bullet)}{dt}$	Scaled time derivative
$\Omega=\frac{\omega}{\omega_0}$	Non-dimensional excitation frequency
$\mu=\frac{c_1}{2m\omega_0}$	Scaled linear damping coefficient
$\nu=\frac{c_2}{m\omega_0}$	Scaled nonlinear damping coefficient
$\beta=\frac{k_1}{m\omega_0^2}=1$	Scaled linear stiffness coefficient
$\delta=\frac{F_p}{m\omega_0^2}$	Scaled parametric excitation amplitude
$\gamma_2=\frac{k_2}{m\omega_0^2}$	Scaled nonlinear quadratic stiffness coefficient
$\gamma_3=\frac{k_3}{m\omega_0^2}$	Scaled nonlinear cubic stiffness coefficient
$\gamma=\gamma_3-\frac{10\gamma_2^2}{9}$	Scaled effective nonlinear stiffness coefficient
$F=\frac{F_d}{m\omega_0^2}$	Scaled direct excitation amplitude

Table S3. Normalized parameter definitions

Here it should be noted that, mass m of the single layer graphene membrane is unknown. Without the exact mass value, optical transduction factors present between the voltage signal measured by the VNA during the experiment and the actual motion of the membrane in physical units cannot be calibrated. Thus, the normalized coefficients shown in table S3 include a linear transduction factor 'κ' for the oscillation amplitude ($x=\kappa V_1$), η for the parametric drive amplitude ($F_\mathrm{p}=\eta V_2$) and λ for the direct drive amplitude ($F_\mathrm{d}=\lambda V_3$). Where V_1, V_2 and V_3 are voltage signals measured in the experiment.

Finally, the equation (S13) is simulated using a pseudo arc length continuation and collocation technique[10] to detect bifurcations and obtain periodic solutions. The simulations are performed as follows:

1. The bifurcation analysis is carried out with the coefficient F as the first continuation parameter and is incremented to the desired value in order to match the experimental direct response.
2. Once the desired value of F is obtained, the parametric drive amplitude δ is used as the second continuation parameter and a value is chosen to replicate the experimental parametric response.
3. After reaching the desired δ value, the analysis is continued with the frequency ratio Ω as the final continuation parameter. This value is spanned around the spectral neighborhood of $\Omega=1$ and $\Omega=2$ in order to obtain the direct and parametric response curves.

S7: Mechanical loss tangent of graphene

In ref.[6] it is shown that the Duffing term γ is proportional to the Young's modulus E:

$$\gamma=CE, \tag{S14}$$

where C is a constant. In case of material damping, a complex Young's modulus can be introduced: $E = E' + iE''$ and the nonlinear stifness term γx^3 near the resonance frequency ω_0, for $x = x_0 e^{-i\omega_0 t}$ becomes:

$$CEx^3 = CE'x^3 + CE''\frac{x^2}{\omega_0}\dot{x} = \gamma x^3 + \nu x^2 \dot{x}. \tag{S15}$$

From this equation it can be seen that the loss tangent $\tan\delta_l = E''/E'$[11] can be calculated by the ratio ν/γ if the resonator is vibrating near its resonance frequency:

$$\tan\delta_l = \frac{\nu}{\gamma}. \tag{S16}$$

References

1. Mahboob, I. & Yamaguchi, H. Piezoelectrically pumped parametric amplification and q enhancement in an electromechanical oscillator. *Appl. Phys. Lett.* **92**, 173109 (2008).
2. Rugar, D. & Grütter, P. Mechanical parametric amplification and thermomechanical noise squeezing. *Phys. Rev. Lett.* **67**, 699 (1991).
3. Aubin, K. *et al.* Limit cycle oscillations in cw laser-driven nems. *J. microelectromechanical systems* **13**, 1018–1026 (2004).
4. Amabili, M. *Nonlinear Vibrations and Stability of Shells and Plates* (Cambridge University Press, 2008).
5. Dolleman, R. J. *et al.* Optomechanics for thermal characterization of suspended graphene. *Phys. Rev. B* **96**, 165421.
6. Davidovikj, D. *et al.* Nonlinear dynamic characterization of two-dimensional materials. *Nat. Commun.* **8**, 1253 (2017).
7. Zaitsev, S., Shtempluck, O., Buks, E. & Gottlieb, O. Nonlinear damping in a micromechanical oscillator. *Nonlinear Dyn.* **67**, 859–883 (2012). URL http://dx.doi.org/10.1007/s11071-011-0031-5. DOI 10.1007/s11071-011-0031-5.
8. Lifshitz, R. & Cross, M. Nonlinear dynamics of nanomechanical and micromechanical resonators. *Rev. nonlinear dynamics complexity* **1**, 1–52 (2008).
9. Nayfeh, A. H. & Mook, D. T. *Non-linear oscillations* (Wiley, 1995).
10. Doedel, E. J. *et al.* Auto 97: Continuation and bifurcation software for ordinary differential equations (1998). URL http://sourceforge.net/project/showfiles.php?groupid=21781.
11. Lakes, R. S. *Viscoelastic Materials*, vol. 1 (Cambridge University Press, New York, USA, 2009).
